# Effectiveness of a Web- and Mobile Phone-Based Intervention to Promote Physical Activity and Healthy Eating in Middle-Aged Males: Randomized Controlled Trial of the ManUp Study

**DOI:** 10.2196/jmir.3107

**Published:** 2014-06-12

**Authors:** Mitch Duncan, Corneel Vandelanotte, Gregory S Kolt, Richard R Rosenkranz, Cristina M Caperchione, Emma S George, Hang Ding, Cindy Hooker, Mohan Karunanithi, Anthony J Maeder, Manny Noakes, Rhys Tague, Pennie Taylor, Pierre Viljoen, W Kerry Mummery

**Affiliations:** ^1^School of Medicine & Public Health, Priority Research Centre in Physical Activity and NutritionFaculty of Health and MedicineUniversity of NewcastleNewcastleAustralia; ^2^Institute for Health and Social Science ResearchCentre for Physical Activity StudiesCentral Queensland UniversityRockhamptonAustralia; ^3^School of Science and HealthUnviersity of Western SydneySydneyAustralia; ^4^Department of Human NutritionKansas State UniversityManhattan, KSUnited States; ^5^School of Health and Exercise SciencesUniversity of British ColumbiaKelowna, BCCanada; ^6^The Australian eHealth Research CentreCSIROBrisbaneAustralia; ^7^School of Computing, Engineering and MathematicsUniversity of Western SydneySydneyAustralia; ^8^Animal, Food and Health SciencesCSIROAdelaideAustralia; ^9^Central Queensland UniversityMackayAustralia; ^10^Faculty of Physical EducationUniversity of AlbertaEdmonton, ABCanada

**Keywords:** physical activity, diet, mobile phone, Web-based, randomized controlled trial

## Abstract

**Background:**

The high number of adult males engaging in low levels of physical activity and poor dietary practices, and the health risks posed by these behaviors, necessitate broad-reaching intervention strategies. Information technology (IT)-based (Web and mobile phone) interventions can be accessed by large numbers of people, yet there are few reported IT-based interventions targeting males’ physical activity and dietary practices.

**Objective:**

This study examines the effectiveness of a 9-month IT-based intervention (ManUp) to improve the physical activity, dietary behaviors, and health literacy in middle-aged males compared to a print-based intervention.

**Methods:**

Participants, recruited offline (eg, newspaper ads), were randomized into either an IT-based or print-based intervention arm on a 2:1 basis in favor of the fully automated IT-based arm. Participants were adult males aged 35-54 years living in 2 regional cities in Queensland, Australia, who could access the Internet, owned a mobile phone, and were able to increase their activity level. The intervention, ManUp, was based on social cognitive and self-regulation theories and specifically designed to target males. Educational materials were provided and self-monitoring of physical activity and nutrition behaviors was promoted. Intervention content was the same in both intervention arms; only the delivery mode differed. Content could be accessed throughout the 9-month study period. Participants’ physical activity, dietary behaviors, and health literacy were measured using online surveys at baseline, 3 months, and 9 months.

**Results:**

A total of 301 participants completed baseline assessments, 205 in the IT-based arm and 96 in the print-based arm. A total of 124 participants completed all 3 assessments. There were no significant between-group differences in physical activity and dietary behaviors (*P*≥.05). Participants reported an increased number of minutes and sessions of physical activity at 3 months (exp(β)=1.45, 95% CI 1.09-1.95; exp(β)=1.61, 95% CI 1.17-2.22) and 9 months (exp(β)=1.55, 95% CI 1.14-2.10; exp(β)=1.51, 95% CI 1.15-2.00). Overall dietary behaviors improved at 3 months (exp(β)=1.07, 95% CI 1.03-1.11) and 9 months (exp(β)=1.10, 95% CI 1.05-1.13). The proportion of participants in both groups eating higher-fiber bread and low-fat milk increased at 3 months (exp(β)=2.25, 95% CI 1.29-3.92; exp(β)=1.65, 95% CI 1.07-2.55). Participants in the IT-based arm were less likely to report that 30 minutes of physical activity per day improves health (exp(β)=0.48, 95% CI 0.26-0.90) and more likely to report that vigorous intensity physical activity 3 times per week is essential (exp(β)=1.70, 95% CI 1.02-2.82). The average number of log-ins to the IT platform at 3 and 9 months was 6.99 (SE 0.86) and 9.22 (SE 1.47), respectively. The average number of self-monitoring entries at 3 and 9 months was 16.69 (SE 2.38) and 22.51 (SE 3.79), respectively.

**Conclusions:**

The ManUp intervention was effective in improving physical activity and dietary behaviors in middle-aged males with no significant differences between IT- and print-based delivery modes.

**Trial Registration:**

Australian New Zealand Clinical Trials Registry: ACTRN12611000081910; https://www.anzctr.org.au/Trial/Registration/TrialReview.aspx?ACTRN=12611000081910 (Archived by WebCite at http://www.webcitation.org/6QHIWad63).

## Introduction

Regular physical activity and healthy eating are key health behaviors that contribute to reducing the risk of chronic disease [1,2]. These behaviors and their impact on health are particularly relevant for Australian males because the majority of Australian males are physically inactive and have poor dietary behaviors [3]. For example, approximately 48% of males are not sufficiently physically active and most males do not meet the recommended intake levels of fruit (54%), vegetables (85%), low-fat dairy (63%), or foods containing high levels of saturated fat and sugar (70%) [3-5]. Males are also less likely to participate in behavioral and information technology (IT)-based interventions compared to females [6-8]. In addition, many males have low levels of health literacy, which is the ability to understand and process health information and use this to assist in changing behaviors [9-11]. Health literacy is an important determinant of health; higher levels of health literacy are associated with engagement in various health-promoting behaviors including physical activity and fruit and vegetable consumption [12]. Improving health literacy has been recognized as key to assisting in improving the overall health of Australian males [13]. The high prevalence of poor health behaviors and low levels of health literacy in males combined with the low levels of engagement by males in many behavioral interventions, particularly IT-based interventions, highlights the need for broad-reaching effective interventions specifically developed for this population.

It is widely acknowledged that Web-based- and/or mobile phone-based interventions (IT-based) provide a delivery method that can be conveniently accessed by a large number of individuals thereby increasing the potential reach relative to other commonly used intervention modes, such as print-based materials [14-16]. IT-based interventions have been used to effectively change physical activity and healthy eating behaviors and are viewed positively by males as an intervention delivery mode [7,17-19]. A broad range of features and components can be implemented in these interventions, including education materials, social interaction/support tools, self-monitoring, and goal-setting features, all of which have been associated with increased behavior change [16,20]. Increased participant use of and engagement with the intervention platform is also associated with greater behavior change [21-24]. Mobile phones and smartphones offer participants greater convenience to access intervention materials, and intervention delivery via smartphone and a website is associated with greater levels of self-monitoring and behavior change when compared to self-monitoring via website only [23]. Therefore, delivery of IT-based interventions using a combination of website and mobile devices may be an effective way to increase participant engagement with the intervention and promote greater behavior change. Despite the potential of IT-based interventions to change physical activity and dietary behaviors, there have been few IT-based interventions that have been specifically developed for and targeted toward males [7,8]. Therefore, the purpose of this study is to examine the effectiveness of a 9-month Web- and mobile phone-based (IT-based) intervention to improve the physical activity and dietary behaviors compared to a print-based intervention [25]. A secondary objective is to compare changes in health literacy between the IT- and print-based intervention groups. It was hypothesized that the IT-based intervention would be more effective in improving outcomes compared to the print-based intervention.

## Methods

### Design

The rationale, design, and methods for the ManUp study, including an outline of the intervention, are described in detail elsewhere [25] and only summarized in brief here. The ManUp study was a 2-arm randomized controlled trial (RCT) with participants randomly allocated to either the IT-based (website and mobile device) intervention arm or a print-based intervention arm. A print-based intervention was selected as the comparison group because they are effective in improving health behaviors [26,27]. All participants received written and verbal explanation of the project requirements before providing consent and provided informed consent before participation in the study. The Central Queensland University (H10/07-131) and the University of Western Sydney Human Research Ethics Committee approved the study (H8605). The study was registered with the Australian New Zealand Clinical Trials Registry (ACTRN12611000081910).

### Participants

Males aged 35 to 54 years who (1) owned a mobile telephone, (2) had access to the Internet, (3) did not have a mobility impairment, (4) resided in the cities of Gladstone or Rockhampton (Queensland, Australia), and (5) were classified as low risk to increase physical activity according to established guidelines were eligible to participate in the study [28]. A combination of online forms and phone contact was used to screen participants for eligibility criteria. To recruit participants, advertisements in local newspapers, trading magazines, face-to-face information sessions with local businesses, and distribution of leaflets and posters to local businesses, medical clinics, and offices of allied health professionals were used. No participant incentives were provided in the study. Participants (N=317) were recruited from October 2010 to September 2011, and following initial screening for inclusion criteria, participants were randomly allocated to 1 of the 2 intervention arms. Because IT-based interventions are less frequently examined in male populations [7,8], the number of participants allocated to the intervention arm was maximized in a 2:1 ratio in favor of the IT-based intervention arm. Randomization lists were generated by one of the authors (MJD) using freely available software [29]. Participants were advised of their group allocation via phone. Participants in the IT-based intervention were emailed details to access the intervention platform, including website uniform resource locator (URL), username, and password; participants in the print-based group provided their mailing address to receive the print-based intervention materials. Participants were blinded to group allocation until after baseline assessments were completed. Given that participants completed the assessment of outcome measures via online survey, nonblinding of researchers to participant group allocation was unlikely to bias outcomes.

### ManUp Intervention

The ManUp study was informed by our reviews of published physical activity and dietary interventions for males, our formative research concerning barriers to physical activity and healthy eating behaviors, and our research regarding males’ preferences for IT-based interventions [7,8,17,18]. Both intervention arms provided participants with the same intervention materials and capacity to self-monitor physical activity and dietary behaviors. The IT-based intervention, however, provided participants with the additional ability to receive automated feedback on their progress toward completing their physical activity and dietary behavior goals (ManUp challenge), as well as the ability to interact with other participants on the website [25]. Additionally, specific components of the IT-based intervention (My Profiles, My Mates, My Groups) were intended to foster social support between participants via commenting on and viewing the progress of others in-line with social cognitive theory. There was little to no interaction between project staff and participants in either intervention arm.

Both interventions arms were provided with educational materials that were specifically designed to present information on the benefits of physical activity and healthy eating and on the volume and type of activity needed to achieve health benefits. Materials provided to participants allowed daily self-monitoring of physical activity and dietary behaviors and highlighted the importance of self-monitoring as a way to change behavior and keep track of the changes made. Participants could record physical activity and dietary behaviors using any metric specified in Table 1. Informed by social cognitive theory and self-regulation theory. ManUp “challenges” were developed to change target behaviors by providing a goal for behavior change, having participants engage in goal setting and self-monitoring behaviors, and also build confidence to make positive changes to behaviors [25,30,31]. An overview of these theories and the role of self-monitoring in changing behavior can be found elsewhere [30-32].

**Table 1:** Description of the ManUp physical activity and healthy eating challenges.

Activity	Light strength (3 weeks)	Mid strength (6 weeks)	Full strength (12 weeks)
Walking	1.5 hours/week or 7500 steps/day	2.5 hours/week or 10000 steps/day	3.5 hours/week or 12000 steps/day
Cycling	1 hours/week or 25 km/week	2 hours/week or 50 km/week	4 hours/week or 100 km/week
Swimming	0.5 hours/week or 1 km/week	1 hours/week or 2 km/week	1.5 hours/week or 3 km/week
Running	0.5 hours/week or 5 km/week	1 hours/week or 10 km/week	2.0 hours/week or 20 km/week
Sport and recreation	0.5 hours/week	1 hours/week	1.5 hours/week
Strengthening	Set 8 exercises 1× set (8-10 reps) 2×/week	Set 8 exercises 2× set (8-10 reps) 2×/week	Set 8 exercises 3× set (8-10 reps) 2×/week
Healthy eating^a^	≥3 healthy eating goals/day	≥5 healthy eating goals/day	≥7 healthy eating goals/day

^a^The ManUp healthy eating goals were: (1) eat 2 servings of fruit, (2) eat 5 servings of vegetables, (3) eat 1 serving of fish, (4) choose whole-grain bread instead of white bread, (5) choose low-fat dairy products, (6) have a soft drink- (soda-) free day, (7) have an alcohol-free day, (8) have an red meat–free day, (9) have an unhealthy snack-free day, and (10) have a fast food–free day.

### ManUp Challenges

The ManUp challenges consisted of 6 physical activity and a multicomponent healthy eating challenge. Each challenge had 3 different “strengths” (light, mid, full), which varied the duration and the amount of activity or healthy eating that males were asked to achieve to complete the challenge. To complete a challenge, participants had to record the required number of minutes/distance/steps for activities or the number of healthy eating goals before the end of the challenge period; failure to do this meant the challenge was not completed. The variation between challenge strengths was intended to provide participants with an appropriate target relative to their current level of physical activity or healthy eating, or to provide a progression toward engaging in higher levels of physical activity or healthy eating. The challenges could be completed in any order preferred by participants and there was no requirement to complete all the different strength challenges or different physical activity challenges. The different activities selected for inclusion were based on those activities frequently performed by Australian males [33]. The ManUp healthy eating challenges were based on achieving a maximum of 10 daily healthy eating goals. These goals were informed by the dietary guidelines for Australian adults that promote dietary diversity and encourage the reduction of the intake of saturated fat, salt, alcohol, and foods that contain added sugars [34]. Although the challenges were informed by the sorts of physical activities frequently participated in by males and the need to promote dietary diversity, they were not intended to promote adherence to public health guidelines for either physical activity or dietary behaviors. Rather, they were designed to increase overall engagement in physical activity and healthy eating. Further details on the types of different physical activities and dietary behaviors targeted, the requirements for each challenge, and supporting educational materials are provided in Table 1 and elsewhere [25].

### Intervention Arms

#### Information Technology–Based Intervention Arm

Upon completing the baseline assessment participants in the IT-based intervention arm received access to the password-protected ManUp website, which had 6 main sections [25]. The 6 sections were:

1. My Profile where participants could review their current challenges, record their progress toward any current challenges, post personal updates to their profile, schedule future activities, and view information on the groups they were a member of and the list of their mates (online friends on the website).
2. My Progress where participants could review their progress toward their current challenges.
3. My Mates where participants could search for online friends and view their mates’ progress. Online friends were limited only to participants allocated to the IT-based intervention; participants could not view an online list of other participants nor were they informed by project staff who else was enrolled in the study because of privacy concerns, but participants could search for other users by entering a name or part of a name into the search tool provided on the website.
4. My Groups where participants could create a group and view the progress of groups they were part of.
5. My Weight, which provided participants with information on the benefits of achieving a healthy weight, and allowed them to record their height, weight, and waist circumference. This section did not allow participants to track these metrics over time; rather, it provided immediate feedback on what category, such as body mass index (BMI) category or waist circumference category, the respective measure was classified as in comparison to established categories for BMI and waist circumference [35].
6. Information Center, which provided educational materials related to physical activity and healthy eating, and the challenges [25].

**Figure 1:**
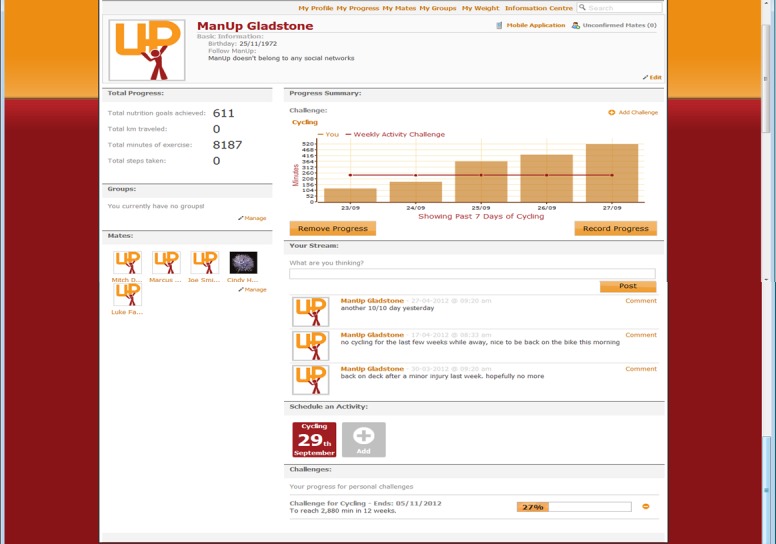
Screenshot of the My Profile section of the ManUp website.

**Figure 2:**
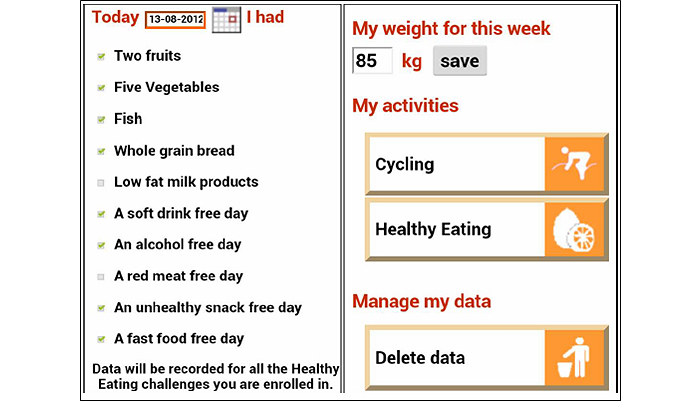
Screenshot of the healthy eating data entry screen of the ManUp app.

**Figure 3:**
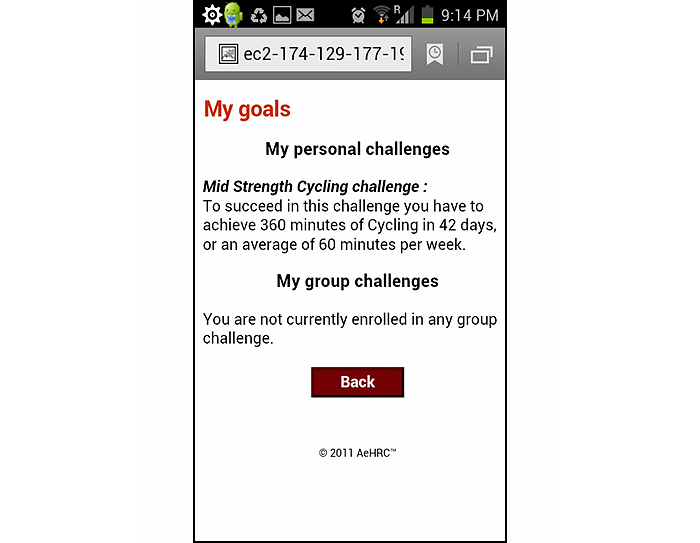
Screenshot of the challenge progress feedback screen of the ManUp app.

As a form of online social support, participants could comment on their mates’ My Profile page. In addition, participants could also challenge their mates to complete a physical activity or healthy eating challenge either in a one-on-one basis, or as part of a larger group. A mobile phone Web application was developed as an additional tool to facilitate quick and convenient recording of progress toward the ManUp challenges. The mobile phone Web application only allowed users to self-monitor behavior and body weight, and to review progress toward challenge completion. Any participant in the IT-based intervention arm who owned a mobile phone capable of accessing the Internet had access to the mobile phone Web application. Figure 1 shows the My Profile section of the website and Figures 2 and 3 display the app data entry screen for healthy eating, the screen showing feedback information on the level of activity needed to complete a particular challenge. Graphical- and text-based feedback of progress toward the completion of a challenge was automatically provided by both the website and app, and was updated based on participant’s self-monitoring entries of physical activity and health eating. Participants were not provided with any detailed instruction on how to use the IT platform or the frequency that it should be used. The initial email providing log-in details suggested they should visit the intervention platform and initiate a ManUp challenge. A short video presenting the main features and functionality of the website was available for viewing on the front page of the website without having to log in to the website. This video was viewed 243 times in total throughout the intervention period.

#### Print-Based Intervention Arm

Participants in the print-based group received a hard-copy booklet that provided the same educational materials (including content from the My Weight section) and ManUp challenges as those provided to participants in the IT-based intervention. Participants in the print-based group were provided with information about using the provided log sheets and could self-monitor progress and/or successful completion of the ManUp physical activity or healthy eating challenges using the log sheets. Participants in the print-based group were not provided with information regarding their peers who were also part of this group. The hard-copy booklet was not collected from participants and no information about the challenges completed or self-monitoring was obtained. Hard-copy booklets were not collected because of logistical reasons and to allow participants to keep a record of their progress to assist in behavior change.

### Outcome Measures

#### Overview

Participants completed online surveys at baseline (0 months), 3 months, and 9 months to assess sociodemographic, behavioral, and health literacy outcomes. Measures of satisfaction with the intervention were also obtained at the 9-month assessment point. All participants received up to 3 phone calls or emails at each assessment point to remind them to complete their assessments.

#### Physical Activity

Physical activity was assessed using the Active Australia Questionnaire, a valid and reliable instrument that is also sensitive to change in physical activity [36-39]. This questionnaire asks participants to report the duration of recreational and transport walking, moderate and vigorous intensity physical activity in the previous week, and the number of times (sessions) they engaged in these activities. Standard scoring protocols were applied to provide 2 outcomes: total minutes of physical activity and the total number of sessions of physical activity [36].

#### Dietary Behaviors

Dietary behaviors were assessed using 19 items adapted from existing instruments used to monitor dietary habits of the Australian population [40,41]. These items have sound psychometric properties [40]. Two separate items assessed the daily number of servings of fruit and vegetables consumed in the past week, response options ranged from zero servings (don’t eat this food) to 10 or more servings. The frequency that red meat, fish, meat products (sausages, salami, meat pies, etc), cooked cereals, soft drinks, chips, takeaway foods, and sweet or savory foods were consumed in the past week were assessed using response options from rarely/never (don’t eat this food) to more than 10 times. The type of milk (whole milk or full cream, reduced fat, soymilk, condensed milk, don’t drink milk) and bread (white, wholemeal, multigrain, rye, sour dough, other, don’t eat bread) usually consumed were also assessed. Three dietary outcomes were created: type of milk consumed (reduced fat vs whole milk), type of bread consumed (higher-fiber wholemeal, multigrain, white with high fiber, sour dough, rye vs white) and an overall index of other dietary behaviors (the dietary score). The dietary score was created by summing the number of servings and number of times the following foods were consumed: fruit, vegetables, red meat, fish, meat products, soft drinks, chips, takeaway (take-out) foods, and sweet and savory foods. Several items were reversed-scored so that higher dietary scores (a better diet) reflected more frequent consumption of healthy food and less frequent consumption of less healthy foods. The dietary score reflected the fact that the ManUp healthy eating challenge focused on maximizing consumption of healthy foods and minimizing consumption of less healthy foods.

#### Health Literacy

Health literacy in relation to physical activity was assessed using the 5 awareness items from the Active Australia Questionnaire [36]. Using a 5-point Likert-type scale from strongly agree to strongly disagree, the items assess awareness of the benefits associated with physical activity participation, and the intensity and duration required to receive health benefits. Dietary behavior literacy was assessed using the Nutritional Literacy Survey, a valid and reliable 28-item instrument that assesses participants’ understanding of the type of foods that promote heart health, and the fat and cholesterol content of different foods and portion sizes [42].

#### Satisfaction

Participant satisfaction with the intervention platform and challenge concept was assessed using 4 items. Using a 5-point scale ranging from strongly agree to strongly disagree, participants indicated if they would like to continue to use the IT- or print-based materials, if the materials (print booklet or IT-based platform) were easy to use, and if they liked the overall concept of the physical activity and healthy eating challenges.

#### Information Technology Platform Usage

Usage of the IT-based platform was measured using in-built tracking software measuring the number of times a participant logged into the Web- and mobile-based platform, made a self-monitoring entry, and the type and number of challenges they initiated and completed.

### Sample Size

Using established methods to estimate sample size [43], the study was powered to detect a 60-minute change in moderate-to-vigorous intensity physical activity per week from baseline to 9 months using an alpha level of .05 and a power level of 90%. Based on this calculation, it was estimated that 197 participants would be required. However, this number was increased to account for the 2:1 allocation of participants in favor of the IT-based intervention arm and the expected dropout rate of participants (45%) [21,44]. A higher dropout rate was used in the current study given the acknowledged difficulty in engaging and retaining males in interventions [7,8]. As a result, the estimated total sample size was 321: 107 allocated to the print-based group and 214 allocated to the IT-based group [25].

### Analysis

Comparisons between groups at baseline were conducted using generalized linear models and chi-square tests. Comparisons between those participants completing all 3 assessment points (completers) and those completing less than 3 assessment points (noncompleters) were made on age, education, physical activity, dietary behaviors, and health literacy using *t* tests (where parametric assumptions were met) or Mann-Whitney *U* for continuous variables, and chi-square tests for categorical variables. Generalized linear mixed models use all available data at each time point allowing participants with missing data at follow-up time points to be retained in the analysis. Therefore, generalized linear mixed models with an unstructured covariance matrix were used to examine change over time and differences between intervention arms in physical activity, dietary behaviors, and health literacy outcomes. All analyses were adjusted for baseline age, occupation, and education because these variables likely impact upon the physical activity and dietary behaviors of males [45]. Outcomes of the generalized linear mixed model analyses are reported as exponentiated coefficients (exp(β)). To explore the impact of missing data, a sensitivity analysis using baseline observation carried forward (BOCF) for participants with missing data at follow-up time points was performed for physical activity, dietary behaviors, and health literacy outcomes; this analysis also adjusted for baseline age, occupation, and education. Comparison of change in physical activity, dietary behaviors, and health literacy with and without BOCF revealed only small differences in the magnitude of these outcomes with the exception of consumption of higher-fiber bread and low-fat milk consumption. For both of these outcomes, the significant time effects present at 3 months in the analysis without BOCF were in the same direction, although not statistically significant in the analysis with BOCF. Given these minor differences, only the results from the analyses without BOCF are reported.

Analyses examining the relationship between usage of the IT platform and change in behavior within the IT-based intervention arm were conducted using generalized linear models adjusted for age, occupation, education, and the baseline level of the outcome examined. The specific model type, link function used for analyses, and the total number of observations included are listed in the footnotes of the relevant tables. All analyses were conducted with SPSS version 20 (IBM Corp, Armonk, NY, USA), followed intention-to-treat principles, and used an alpha level of .05.

## Results

### Participants

**Figure 4:**
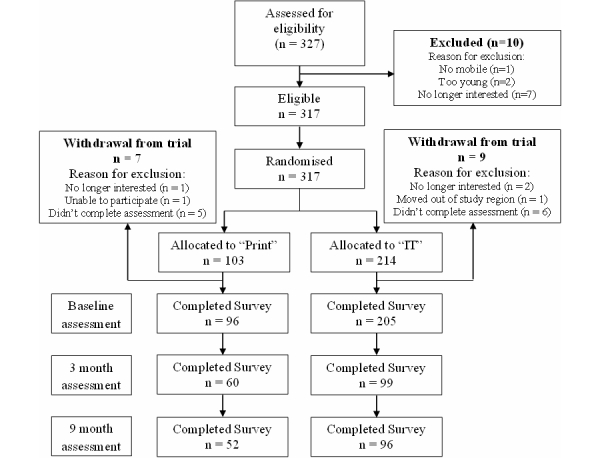
Flow of participants through the study.

The flow of participants through the study, including the number of participants completing each assessment, is provided in Figure 4. A total of 327 males expressed an interest in participating in the study: 10 males were excluded because they did not satisfy eligibility criteria or were no longer interested in participating, 317 males were randomized to an intervention arm of which 16 withdrew from the study before completing a baseline assessment (Figure 4). A total of 301 participants completed the baseline assessment, 159 completed the 3-month assessment, 148 completed the 9-month assessment, and 125 completed all 3 assessment points. No significant differences were observed between those who completed 3 assessment points (completers) compared to those completing less than 3 assessment points (noncompleters) on baseline minutes and sessions of physical activity, dietary score, and type of bread consumed (data not shown). Completers were significantly different from noncompleters in terms of age (mean 45.11, SE 0.51 vs mean 43.32, SE 0.44; *P*=.01), the proportion working in professional level occupations (66.4% vs 49.4%; *P*=.03), the proportion reporting a university-level education (62.6% vs 41.5%; *P*=.002), the proportion reporting to consume low-fat milk (66.4% vs 49.7%; *P*=.01), the proportion reporting that at least 20 minutes of vigorous intensity physical activity 3 times per week is essential to improve health (35.8% vs 64.2%; *P*=.01), and nutritional literacy (mean 25.53, SE 0.16 vs mean 24.98, SE 0.16; *P*=.02).

There were no significant baseline differences between the print-based arm and the IT-based arm for any demographic behavioral and health literacy variable, with the exception that there were fewer participants who agreed that 30 minutes of physical activity is enough to improve health in the IT-based arm compared to the print-based arm (Table 2). In-line with the eligibility criteria, all participants owned a mobile phone and 151 (73.2%) of the IT-based intervention owned a phone that could access the Internet.

**Table 2:** Sociodemographic, anthropometric, and behavioral characteristics of participants at baseline.

Participant characteristic		Print-based n=96	IT-based n=205	*P*
Age (years), mean (SE)		43.84 (0.59)	44.17 (0.41)	.66
**Occupation, n (%)**				.64
	Professional	52 (54.2)	118 (57.6)	
	White collar	8 (8.3)	16 (7.8)	
	Blue collar	23 (24.0)	37 (18.0)	
	Other	13 (13.5)	34 (16.6)	
**Education level, n (%)**				.72
	Secondary school or less	20 (20.8)	45 (22.0)	
	TAFE^a^	25 (26.0)	61 (29.8)	
	University	51 (53.1)	99 (48.3)	
**Self-reported BMI, n (%)**				.46
	Healthy weight	13 (13.5)	19 (9.3)	
	Overweight	41 (42.7)	85 (41.5)	
	Obese	42 (43.8)	101 (49.3)	
Self-reported minutes of physical activity/week, mean (SE)		277.94 (29.15)	286.12 (24.72)	.83
Self-reported sessions of physical activity/week, mean (SE)		4.0 (1.0, 8.0)	4.0 (1.0, 7.0)	.95
Dietary score, median (IQR)		52.0 (46.0, 56.75)	52.0 (47.0, 57.0)	.33
Higher-fiber bread, n (%)		93 (57.0)	195 (68.2)	.06
Low-fat milk, n (%)		87 (57.5)	182 (56.0)	.83
**Physical activity literacy (% agree), n (%)**				
	≥30 min/day improves health	79 (82.3%)	144 (70.2%)	.03
	30 min brisk walking improves health	79 (82.3%)	153 (74.6%)	.14
	20 min of vigorous activity 3 times/week is essential	54 (56.3%)	139 (67.8%)	.05
	10-min blocks of activity are okay	52 (54.2%)	106 (51.7%)	.69
	Moderate activity can improve health	87 (90.6%)	177 (86.3%)	.29
Nutritional literacy, median (IQR)		25 (24, 26)	26 (24, 27)	.66

^a^Technical and further education (TAFE) is a provider of vocational nonbachelor education up to level of advanced diploma.

### Change in Physical Activity and Dietary Behaviors

There were no significant between-group differences or group×time interaction effects in any of the physical activity and dietary behaviors examined; however, significant main effects for time were observed (Table 3). Self-reported minutes (3 months: exp(β)=1.45, 95% CI 1.09-1.95; 9 months: exp(β)=1.55, 95% CI 1.14-2.10) and sessions of physical activity (3 months: exp(β)=1.61, 95% CI 1.17-2.22; 9 months: exp(β)=1.51, 95% CI 1.15-2.00) were significantly higher at 3 and 9 months compared to baseline in both groups. Dietary scores were significantly higher (improved) at both 3 and 9 months compared to baseline (3 months: exp(β)=1.07, 95% CI 1.03-1.11; 9 months: exp(β)=1.10, 95% CI 1.05-1.13) in both groups. A significantly higher proportion of participants reported consuming higher-fiber bread (exp(β)=2.25, 95% CI 1.29-3.92) and low-fat milk (exp(β)=1.65, 95% CI 1.07-2.55) at 3 months compared to baseline in both groups; consumption of higher-fiber bread and low-fat milk were not significantly higher at 9 months compared to baseline in both groups.

**Table 3:** Comparison of self-reported measured health behaviors between intervention groups over the intervention period.

Health behavior		exp(β) (95% CI)	Model effects, *P*		
			Group	Time	Group×time
**Self-report minutes of physical activity per week** ^a^			.60	<.001	.66
	IT-based vs print-based^b^	1.03 (0.78-1.36)			
	3 vs 0 months^b^	1.45 (1.09-1.95)			
	9 vs 0 months^b^	1.55 (1.14-2.10)			
**Self-report sessions of physical activity per week** ^a^			.32	<.001	.55
	IT-based vs print-based^b^	0.97 (0.75-1.25)			
	3 vs 0 months^b^	1.61 (1.17-2.22)			
	9 vs 0 months^b^	1.51 (1.15-2.00)			
**Dietary score** ^c^			.68	<.001	.09
	IT-based vs print-based^b^	1.02 (0.98-1.06)			
	3 vs 0 months^b^	1.07 (1.03-1.11)			
	9 vs 0 months^b^	1.10 (1.05-1.13)			
**Higher-fiber bread** ^d^			.05	<.001	.92
	IT-based vs print-based^b^	1.60 (0.94-2.71)			
	3 vs 0 months^b^	2.25 (1.29-3.92)			
	9 vs 0 months^b^	1.89 (0.99-3.60)			
**Low-fat milk** ^e^			.54	.002	.90
	IT-based vs print-based^b^	0.88 (0.52-1.49)			
	3 vs 0 months^b^	1.65 (1.07-2.55)			
	9 vs 0 months^b^	1.41 (0.92-2.17)			

^a^Model (negative binomial with log link) included age, education level, and occupational classification as covariates. Number of observations=616.

^b^Reference category for comparison.

^c^Model (negative binomial with log link) included age, education level, and occupational classification as covariates. Number of observations=608. This outcome was examined as the change in the total number of times the food was consumed and the servings of a food.

^d^Model (binomial with logit link) included age, education level, and occupational classification as covariates. Number of observations=587. This outcome was examined as the change in the proportion of participants consuming higher-fiber bread.

^e^Model (binomial with logit link) included age, education level, and occupational classification as covariates. Number of observations=542. This outcome was examined as the change in the proportion of participants consuming low-fat milk.

### Change in Health Literacy

A significantly lower proportion of participants in the IT-based intervention arm reported agreeing that 30 minutes of physical activity per day improves health compared to the print-based arm (exp(β)=0.48, 95% CI 0.26-0.90); there were no significant time or group×time interaction effects for this outcome (Table 4). A significantly higher proportion of participants in the IT-based intervention arm reported agreeing that 20 minutes of vigorous intensity physical activity performed 3 times per week is essential to improve health (exp(β)=1.70, 95% CI 1.02-2.82). There were no significant group or group×time interaction effects for the proportion of participants reporting that blocks of a minimum of 10 minutes physical activity are acceptable to acquire health benefits; however, a significantly higher proportion of participants reported agreeing with this statement at 9 months compared to baseline in both groups (exp(β)=2.52, 95% CI 1.28-4.94). No other statistically significant differences were observed in physical activity and nutrition literacy (Table 4).

**Table 4:** Comparison of health literacy outcomes between intervention groups over the intervention period.^a^

Health literacy outcome		exp(β) (95% CI)	Model effects, *P*		
			Group	Time	Group×time
≥**30 minutes/day improves health** ^b^			.17	.11	.28
	IT-based vs print-based^c^	0.48 (0.26-0.90)			
	3 vs 0 months^c^	1.02 (0.50-2.09)			
	9 vs 0 months^c^	1.37 (0.65-2.89)			
**30 minutes brisk walking improves health** ^b^			.91	.01	.13
	IT-based vs print-based^c^	0.63 (0.34-1.16)			
	3 vs 0 months^c^	1.33 (0.58-3.06)			
	9 vs 0 months^c^	1.51 (0.60-3.81)			
**20 minutes of vigorous activity 3 times/week is essential** ^b^			.01	.99	.88
	IT-based vs print-based^c^	1.70 (1.02-2.82)			
	3 vs 0 months^c^	0.96 (0.49-1.87)			
	9 vs 0 months^c^	1.08 (0.57-2.04)			
**10-minute blocks of activity are okay** ^b^			.33	.001	.58
	IT-based vs print-based^c^	0.89 (0.54-1.45)			
	3 vs 0 months^c^	1.51 (0.83-2.72)			
	9 vs 0 months^c^	2.52 (1.28-4.94)			
**Nutrition literacy** ^d^			.78	.44	.51
	IT-based vs print-based^c^	1.01 (0.99-1.03)			
	3 vs 0 months^c^	1.01 (0.99-1.03)			
	9 vs 0 months^c^	1.01 (0.97-1.05)			

^a^Analysis of change in the physical activity literacy outcome of “moderate physical can improve health” is not reported as there was insufficient variation in the outcome to allow the model to be accurately estimated. The proportion of participants agreeing with this statement at each time point in each group is: Baseline: IT-based=86.3%, print-based=90.6%; 3 months: IT-based=88.9%, print-based=86.7%; 9 months: IT-based=100.0%, print-based=95.8%.

^b^Model (binomial with logit link) included age, educational level, and occupational classification as covariates. Number of observations=608.

^c^Reference category for comparison.

^d^Model (negative binomial with log link) included age, educational level, and occupational classification as covariates. Number of observations=608.

### ManUp Challenges

**Figure 5:**
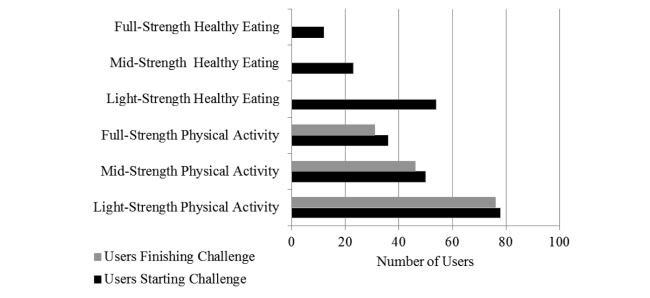
Number of participants in the IT-based intervention who started and completed ManUp challenges.

Because of the difficulty in obtaining records in logbook usage and challenge completion in the print-based intervention arm, data on the usage of ManUp challenges is only reported for the IT-based intervention arm. Figure 5 demonstrates the number of participants in the IT-based intervention arm who started and completed light-, mid-, or full-strength physical activity and healthy eating challenges over the 9-month intervention period. A higher number of participants initiated physical activity challenges compared to the healthy eating challenges, and no participants completed a healthy eating challenge—this was because of participants not completing the required number of healthy eating goals in the specified time period (3-, 6-, or 12-week challenge duration depending on challenge strength selected); thus, they did not complete the challenge. When examining the number of challenges initiated and completed over the 9-month period by all IT-group participants, light-strength physical activity challenges were the most frequently selected (n=147) and completed (n=137), followed by mid-strength physical activity challenges (initiated: n=80; completed: n=68), and full-strength physical activity challenges (initiated: n=69; completed: n=53). Healthy eating challenges were initiated at a lower frequency compared to physical activity challenges but followed a similar pattern; light-strength healthy eating challenges were the most frequently selected (initiated: n=60; completed: n=0), followed by mid-strength healthy eating challenges (initiated: n=28; completed: n=0), and full-strength healthy eating challenges (initiated: n=14; completed: n=0).

**Figure 6:**
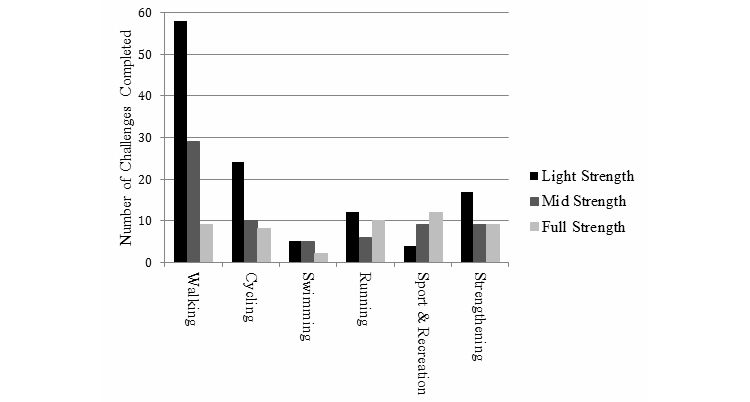
Number of physical activity challenge types completed by challenge strength.

**Figure 7:**
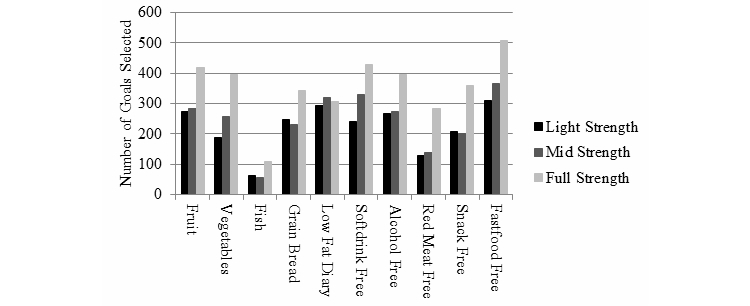
Number of healthy eating goals selected by challenge strength (based on goals logged via the website only).

Information on the type of physical activity challenges selected by participants is provided in Figure 6. Walking was the most frequently selected challenge type, followed by cycling and strengthening activities. Figure 7 shows the number of different health eating goals selected; fast food–, soft drink–, and alcohol-free days and eating 2 pieces of fruit were the most frequently selected goals across all challenges.

### Information Technology Platform Usage

**Figure 8:**
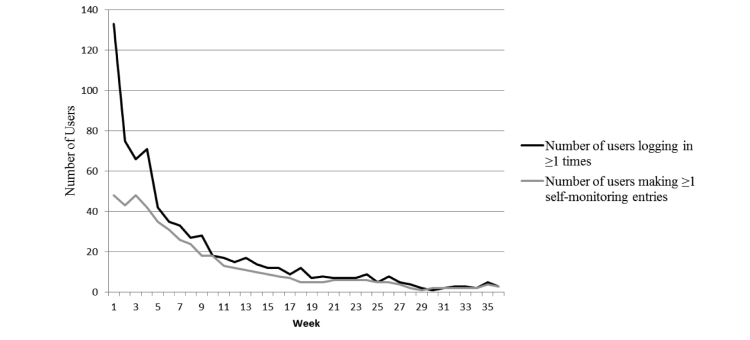
Participant usage of the IT-based intervention platform each week over the intervention period.

The median number of log-ins to the IT platform per week at 3 months and 9 months was 2.00 (IQR 6.00) and 2.00 (IQR 6.50), respectively; the average number of log-ins to the IT platform at these same time periods was 6.99 (SE 0.86) and 9.22 (SE 1.47). Median number of self-monitoring entries per week at 3 months and 9 months was 1.00 (IQR 20.0) and 1.00 (IQR 21.5), respectively; the average number of self-monitoring entries at 3 months and 9 months was 16.69 (SE 2.38) and 22.51 (SE 3.79), respectively. Participants who logged in 2 or more times in the first 3 months of the intervention made significantly more self-monitoring entries (median 18.00, IQR 38.00) compared to participants logging in less than 2 times (median 0.00, IQR 0.00; *U*=1195.50, *P*<.001). Figure 8 shows the number of users logging in at least once and making at least 1 self-monitoring entry each week over the intervention period. Following the initial reduction in usage between week 1 and week 3, usage continued to decline throughout the intervention period. No measure of usage of the IT-based platform was associated with any of the physical activity or dietary behaviors examined (Table 5). Use of the Mate feature and posts to update their progress was low; 21 participants used the Mate feature and no participants using this feature had more than 1 mate on the platform; 36 participants used the post feature (minimum=1 post, maximum=26 posts). Minimal reports of bugs or errors (<10) on the IT-based platform were received during the intervention period via the “report a bug” feature. Of the 33 participants who completed survey items regarding satisfaction of the print materials, 33.3% (11/33) agreed or strongly agreed that they would like to continue to use the ManUp booklet in the future, and 75.8% (25/33) agreed or strongly agreed that it was easy to use. Of the 60 participants who completed survey items regarding satisfaction of the website, 25.0% (15/60) of participants agreed or strongly agreed that they would like to continue to use the website and 85.0% (51/60) reported it was easy to use. Of the 139 participants who completed items regarding satisfaction with the concept of the physical activity and healthy eating challenges, 60.4% (29/48) and 52.7% of participants (48/91) in the print and IT-based groups, respectively, reported satisfaction with the physical activity challenge with no significant differences between groups (χ^2^_1_=0.7, *P*=.34). There were no differences in the proportion of participants in the print (60.4%, 29/48) and IT-based groups (48.4%, 44/91) who reported being satisfied with the healthy eating challenge (χ^2^_1_=1.8, *P*=.18)

**Table 5:** Associations between IT-platform usage and self-reported physical activity and dietary behaviors.

Health behavior		Number of log-ins, exp(β) (95% CI)	Number of self-monitoring entries, exp(β) (95% CI)	Model effects, *P*	
				Number of log-ins	Number of self-monitoring entries
**Self-report minutes of physical activity per week** ^a^					
	3 months	1.00 (0.98-1.01)	1.00 (0.997-1.01)	.43	.38
	9 months	1.00 (0.99-1.00)	1.00 (1.00-1.01)	.25	.10
**Self-report sessions of physical activity per week** ^b^					
	3 months	0.99 (0.98-1.01)	1.01 (1.00-1.01)	.19	.05
	9 months	1.00 (0.99-1.00)	1.00 (1.00-1.01)	.41	.16
**Dietary score** ^c^					
	3 months	1.00 (1.00-1.00)	1.00 (0.99-1.00)	.65	.76
	9 months	1.00 (1.00-1.00)	1.00 (1.00-1.00)	.11	.63
**Higher-fiber bread**					
	3 months^d^	1.03 (0.93-1.13)	1.02 (0.99-1.05)	.59	.25
	9 months^e^	—	—	—	—
**Low-fat milk** ^f^					
	3 months	0.97 (0.90-1.04)	1.01 (0.98-1.04)	.33	.71
	9 months	1.00 (0.98-1.03)	1.00 (0.99-1.01)	.71	.54

^a^Model (Tweedie with log link) included age, educational level, occupational classification, access to the mobile platform, and baseline minutes of physical activity as covariates. 3 months: n=101; 9 months: n=100.

^b^Model (negative binomial with log link) included age, educational level, occupational classification, access to the mobile platform, and baseline sessions of physical activity as covariates. 3 months: n=101; 9 months: n=100.

^c^Model (Tweedie with log link) included age, educational level, occupational classification, access to the mobile platform, and baseline dietary score as covariates. 3 months: n=99; 9 months: n=96.

^d^Model (binomial with logit link) included age, educational level, occupational classification, access to the mobile platform, and baseline bread consumption as covariates. Number of participants=93.

^e^Results are not reported for this time point as the model had partial or complete separation and parameters could not be reliably estimated.

^f^Model (binomial with logit link) included age, educational level, occupational classification, access to the mobile platform, and baseline milk consumption as covariates. 3 months: n=82; 9 months: n=80.

## Discussion

This study examined the relative effectiveness of the ManUp intervention materials delivered by an IT-based intervention platform compared to a print-based intervention to improve middle-aged males’ physical activity and dietary behaviors, and health literacy of these behaviors. Analyses revealed significant improvements over time in self-reported minutes and sessions of physical activity and self-reported overall dietary behaviors in both groups. These changes did not significantly differ between participants receiving access to the IT- or print-based intervention materials. Three components of physical activity literacy changed during the intervention period. First, a lower proportion of participants in the IT-based intervention arm reported agreeing that 30 minutes of physical activity per day is enough to improve health. Second, a higher proportion of participants in the IT-based intervention arm reported agreeing that 20 minutes of vigorous intensity physical activity 3 times per week is necessary to improve health. Finally, a higher proportion of participants from both intervention arms reported agreeing that accumulating physical activity in blocks of a minimum 10 minutes are acceptable to improve health at the 9-month assessment point compared to baseline. Nutrition literacy did not change over time or between intervention arms.

Print- and IT-based interventions have been shown to be effective in improving physical activity, dietary behaviors, or both behaviors in male populations in earlier research [19,46-49]. These studies demonstrated positive intervention effects on target behaviors for time periods ranging between 14 weeks and 12 months [19,48]. Consistent with this literature, the current study demonstrated positive changes in both physical activity and dietary behaviors over a 9-month period. Notwithstanding the limitations of the self-report data in the current study, the sustained nature of the changes is encouraging given that poor physical activity and dietary behaviors are prevalent and significant contributors to chronic disease risk in male populations [3-5,13]. Most previous research has targeted only 1 of these behaviors and comparatively fewer interventions aimed at males have targeted both behaviors simultaneously [7,8]. As such, outcomes of the ManUp study contribute to the evidence that it is feasible to target and significantly improve multiple health behaviors simultaneously in various populations [50-52]. The ability of the intervention platform to improve physical activity and dietary behaviors and the lack of differences between the delivery modes suggests that IT-based approaches are useful to improve males’ engagement in these behaviors. This observation is useful given the large number of males engaging in poor levels of physical activity and dietary behaviors and the potential reach of IT-based interventions relative to other delivery modes. However, as others have noted, the challenge lies in attracting and engaging individuals to the platform [15,53], particularly males who are an acknowledged difficult group to engage in these types of interventions [7,8].

An advantage of IT-based platforms is that participant engagement and usage can be monitored throughout the intervention period. Figure 8 shows a modest level of engagement and usage in the initial weeks of the intervention period followed by a steady decline over time. The modest level of initial usage may be related to the minimal instruction provided to participants on usage of the platform; however, this was intended to reflect the level of instruction provided in other publicly available websites. Platform usage rates may also be related to the levels of participant satisfaction reported which were not as high as expected given the materials and intervention platform were developed specifically for this population. However, given the limited number of participants who completed participant satisfaction survey items these results should be interpreted cautiously. Analysis of the frequency of user log-in and self-monitoring entries revealed no statistically significant association with behavior at either the 3- or 9-month assessment points. Although the pattern of declining participant usage over time is similar to that of previous studies [45,54], the absence of an association with behavior change is not [22-24,45,55]. The lack of association could be because of a lack of statistical power or the measures of engagement and usage applied in the current study failed to capture real participant engagement with the platform. Donkin et al [24] suggested that more in-depth analysis of participant engagement and platform usage is required to understand the relationship between engagement and behavior change; the findings of this study support the assertion that simplistic measures of usage may not be the strongest predictors of behavior change. Alternatively, it may be that participants received an adequate exposure to intervention materials to promote behavior change during their initial use of the platform. The suggestion that an individual’s behavior can be changed following a single exposure to an intervention is supported by previous studies [50,56,57]. Several interventions now seek to maintain and increase greater engagement through various IT-based strategies to foster greater or more sustained behavior change, and evaluation of these strategies to prolong user engagement are in their infancy [45,58]. Given the consistently reported declines in platform usage and engagement over time [15,45], and evidence of effectiveness of repeated interventions delivered in a booster style [59,60], comparing the effectiveness of these 2 intervention approaches to change behavior may contribute to understanding the most effective way to change behavior.

Potential reasons for low engagement, usage, and satisfaction could be a mismatch between participants’ expectations of the intervention and intervention reality. For example, process evaluation of participants in this trial revealed that many wanted prescriptive and personalized information and feedback on their progress [61]. Participants also expressed a desire to transfer the print-based intervention to online platforms to increase accessibility, and for the ManUp app to be usable without requiring an Internet connection [61]. As such, it appears that participants desired an increased accessibility to content above that provided by the current intervention platform. These issues may have adversely impacted engagement and satisfaction. Managing the user expectations by providing a flexible intervention platform that is highly accessible may improve platform usage above that observed in the current trial [61].

One strategy intended to promote prolonged engagement is social interaction among participants [58]. In the current study, 10% of participants had an online friend or mate, and website statistics showed that all these participants had no more than 1 mate. The number of participants with online friends is slightly higher than that reported in a subsample of users of the 10,000 Steps website (4.3%), yet use of online friends appears to be low by participants of health behavior change interventions [22]. Low use of this feature in the current study could be due to several factors, including a lack of awareness of this feature on the intervention platform, the fact the platform required users to search by name for a mate without knowing who else was on the platform, a reluctance to befriend individuals online when they are not “real-life” friends, and the limited number of participants on the website (n=205) compared to other online social networking sites (eg, Twitter, Facebook). The study did not assess if participants knew the identity of other participants also enrolled in the study. These issues should be considered in the design of future interventions seeking to implement a social support/interaction feature. Some of these restrictions were imposed to preserve the integrity of the RCT design, which poses interesting design issues for future studies seeking to evaluate the effectiveness of social interaction within RCT designs. These issues include how to foster online social interaction between individuals who do not know one another in real life, or allowing study participant’s real-life friends to use the platform and maintain the integrity of the trial.

The ManUp challenges allowed participants to select from a range of different challenges that varied in the length of challenge and the amount of the behavior to be performed, and analysis of the challenges selected by participants revealed some interesting results. Figure 5 shows that light-strength challenges were the most frequently initiated challenges for both physical activity and dietary behaviors. This may be a function of participants seeking to try a new activity or dietary change to build confidence in their ability to change before committing to longer-term change [25]. This aligns with behavior change theory, which indicates that successfully completing a task is useful in building confidence to complete subsequent tasks [30]. For the physical activity challenges, participant data indicated that some users selected a variety of different challenge activities, which may be one way users sought to introduce variety to their physical activity regime to maintain interest over a longer time period. The most frequently completed challenge was walking; this is interesting because it has been suggested that walking does not appeal to males in this age range [62]. Yet this does not seem to be the case in the current study, nor does it appear to be mirror broader data for Australian males that indicate walking is the most popular recreational activity engaged in [33]. Figure 7 shows the number of each healthy eating goal completed by challenge strength. The higher number of full-strength goals completed reflects the longer duration and higher number of goals required for this challenge strength. Fast food-, alcohol-, and soft drink-free days and consuming 2 pieces of fruit were the 4 most frequently completed goals overall, which partly aligns with the objective of the healthy eating challenges to incorporate positive changes to dietary habits to improve overall dietary quality. That no healthy eating challenges were completed indicates either poor compliance with implementing changes or that the way in which a challenge needed to be completed (specific number of goals over a specified time period) could be improved. ManUp challenges were designed to provide ready-made targets for males, yet some males expressed a desire to have greater flexibility in setting their own goals and recording progress in their preferred metric [61]. Adopting this approach may have increased the completion rate of the challenges.

Approximately three-quarters of participants in the IT-based arm owned a mobile phone that allowed them to access the Internet and, therefore, the mobile component of the intervention. This is higher than previous reports in Australia, and is likely to continue to increase as ownership of Internet-capable mobile devices continues to increase [63]. Yet only 21.8% of participants in the IT-based intervention who had access actually logged in to the mobile phone platform. The low-level usage of the mobile phone component may be related to its limited functionality. Limiting functionality was a conscious design decision to maximize potential use across a wide variety of mobile phones, including some older mobile phones that did not allow the navigation and functionality of newer smartphone devices. Interviews with intervention participants indicated that needing to be connected to the Internet to use the mobile app was a limiting factor for some users and this may have also contributed to the low usage [61]. Recent growth in and expected growth of smartphone sales and ownership, self-monitoring devices (ie, Fitbit, Jawbone), and willingness of some males to use smartphone technology [17,63,64] will allow future interventions to take advantage of the greater functionality offered by these devices including the ability to perform self-monitoring via installed apps without the device/app requiring an Internet connection.

Health literacy allows individuals to use and apply knowledge to process information and inform decisions concerning their health and is identified as a priority for males [13]. ManUp educational materials were designed to provide clear and concise information on the rationale for changing physical activity and dietary behaviors and how to achieve these changes consistent with formative research and behavior change theory [18,25,30]. Study outcomes show mixed support for the effectiveness of the materials to improve physical activity and nutritional literacy. Nutrition literacy remained unchanged throughout the study and may have been impacted by the moderately high level of nutritional literacy at baseline [42]. Dietary educational materials provided information on the amount and frequency that particular food groups should be eaten consistent with national guidelines and the benefits of consuming the particular food group and an overall healthy diet [25,34]. Although the nutritional literacy scale assessed some components addressed by educational materials (eg, required servings of fruit and vegetables, high-sugar foods contain a high number of kilojoules), other components were much broader (eg, how to prevent food poisoning from eggs, cost and weed control methods of organic farms). As such, the discrepancy between the educational materials provided in the intervention and the instrument used to assess nutritional literacy may have limited the ability to detect change in this construct. Alternatively, participants may not have read these educational materials or preferred a more prescriptive approach to the dietary information provided [61]. Baseline physical activity literacy was lower than previously reported, particularly regarding knowledge that blocks of a minimum of 10 minutes of activity are acceptable to improve health [65]. This was the only measure to significantly change over time with a significantly higher number of participants agreeing with this at 9 months (68.9%) compared to baseline (52.5%). Changes in the proportion of participants agreeing that moderate intensity physical activity can improve health were not statistically examined because more than 95% participants agreed with this statement at 9 months, which resulted in insufficient variability for the analysis to take place (Table 4). Although not statistically examined, this may be viewed as an improvement in one component of physical activity literacy. The significant between-group differences in the proportion of participants agreeing that vigorous intensity physical activity performed 3 times per week is essential for health and that 30 minutes of moderate intensity physical activity per day can improve health are likely because of differing levels between groups at baseline. The reasons for the baseline differences between intervention groups on the proportion who agreed that 30 minutes of moderate intensity physical activity per day can improve health are unknown, particularly because qualitative research indicates that males have a good knowledge of the volumes and frequencies of physical activity needed to improve health [18,66]. Based on these observations, the ManUp education materials may have been more effective at improving physical activity literacy of participants compared to nutritional literacy.

Males are acknowledged as a hard-to-reach population in the health behavior intervention literature and this is reflected in the low recruitment rate in this study (approximately 27 participants per month of recruitment). IT-based interventions frequently report low participant retention rates, and in this study the overall retention rate at 9 months was 49.2% with a lower retention rate in the IT-based group (46.8%) compared to the print-based group (54.2%, *P*=.24). Low retention rates in IT-based studies are an acknowledged issue and the level of retention in this study is comparable to previous intervention studies [67-70]; as such, the low retention rate may not be an issue specific to the target population. The low retention rate limited the number of observations available for analysis over time; however, analytic methods using all available data were employed to minimize the impact of this potential limitation. Consistent with study objectives to improve regular engagement in physical activity and healthy eating, the ManUp study did not focus on weight loss during recruitment which previous studies have promoted in their recruitment [47,48]. This may have contributed to lower than expected recruitment rates given the prevalence of overweight and obesity in males.

Because of logistical constraints, it was not possible to assess usage of the print-based materials and self-monitoring behavior of participants in this intervention arm. The lack of this usage data prohibits between-group comparisons of usage and behavior change, which may contribute to better understanding the relationship between platform usage and behavior change; this is a limitation of the study. Other limitations of the study include a reliance on self-report measures. Although a subsample of participants (n=91) were provided with accelerometers to objectively measure physical activity [25], poor participant compliance with measurement protocols resulted in too few participants providing valid data for meaningful analysis and these data are not reported in this paper. The usage rate of the mobile component and use and functionality aspects of the Mate feature are also limitations of the current study.

This study evaluated the effectiveness of an intervention delivered by IT- and print-based materials to promote self-monitoring of physical activity and dietary behaviors and health literacy of these behaviors. Although study outcomes show mixed support for the intervention to change health literacy, IT- and print-based modes were effective in improving physical activity and dietary behaviors in middle-aged males with no differences between delivery modes. This suggests both may be useful intervention delivery modes.

## Multimedia Appendix 1

CONSORT-EHEALTH checklist V1.6.2 [71].

## Abbreviations

BMI: body mass index
BOCF: baseline observation carried forward
exp(β): exponentiated coefficient
IT: information technology
RCT: randomized controlled trial
URL: uniform resource locator
TAFE: Technical and further education
