# Supplementary material for: Effectiveness of a Web- and Mobile Phone-Based Intervention to Promote Physical Activity and Healthy Eating in Middle-Aged Males: Randomized Controlled Trial of the ManUp Study
Source: J Med Internet Res. 2014 Jun 12;16(6):e136. doi: 10.2196/jmir.3107 (PMC4090375; doi:10.2196/jmir.3107)
Supplement: Supplementary file 1 [file jmir_v16i6e136_app1.pdf]

CONSORT-EHEALTH Checklist V1.6.2 Report

(based on CONSORT-EHEALTH V1.6), available at [http://tinyurl.com/consort-ehealth-v1-6].

3107

Date completed

11/20/2013 4:08:26

by

Mitch J Duncan

A Randomised Controlled Trial examining the effectiveness of a web- and mobile phone-based intervention to promote physical activity and healthy eating in middle-aged males: Outcomes of the ManUp Study'

TITLE

1a-i) Identify the mode of delivery in the title

" web- and mobile phone-based intervention "

1a-ii) Non-web-based components or important co-interventions in title

web- and mobile phone-based intervention '

1a-iii) Primary condition or target group in the title

middle-aged males'

ABSTRACT

1b-i) Key features/functionalities/components of the intervention and comparator in the METHODS section of the ABSTRACT

Participants were randomized into either an IT-based or print-based intervention arm on a 2:1 basis in favour of the IT-based arm. The intervention, ManUp, was informed by Social Cognitive and Self Regulation Theories and was specifically designed to target males. Educational materials were provided and self-monitoring of physical activity and nutrition behaviours was promoted. Intervention content was the same in both intervention arms, only the delivery mode differed, and content could be accessed throughout the 9-month study period. '

1b-ii) Level of human involvement in the METHODS section of the ABSTRACT

Participants were randomized into either an IT-based or print-based intervention arm on a 2:1 basis in favour of the fully automated IT-based arm. '

1b-iii) Open vs. closed, web-based (self-assessment) vs. face-to-face assessments in the METHODS section of the ABSTRACT

Participants, recruited offline (e.g. newspaper adds)'
'... were measured using online surveys at baseline, 3 months and 9 months.'

This study was not blinded, given the differences between groups (IT vs Print) there was no way of blinding in what groups participants were. Because it is so obvious there is no need in mentioning this in the abstract

1b-iv) RESULTS section in abstract must contain use data

A total of 301 participants completed baseline assessments, 205 in the IT-based arm and 96 in the print-based arm. A total of 124 participants completed all three assessments.'

'The average number of logins to the IT-platform at 3 and 9 months was 6.99 (SE = 0.86) and 9.22 (SE = 1.47), respectively. The average number of self-monitoring entries at 3 and 9 months was 16.69 (SE = 2.38) and 22.51 (SE = 3.79), respectively.'

1b-v) CONCLUSIONS/DISCUSSION in abstract for negative trials

Not applicable for this study: outcomes did change!

INTRODUCTION

2a-i) Problem and the type of system/solution

Approximately 48% of males are not sufficiently physically active, and the majority of males do not meet the recommended intake levels of fruit (54%), vegetables (85%), consumption of low-fat dairy (63%) and consumption of foods containing high levels of saturated fat and sugar (70%). '

'Web-based- and/or mobile phone-based interventions (IT-based) provide a delivery method that can be accessed by a large number of individuals. IT-based interventions have been used to effectively change physical activity and healthy eating behaviours and are viewed positively by males as an intervention delivery mode [9-12]. '

2a-ii) Scientific background, rationale: What is known about the (type of) system

Delivery of IT-based interventions using a combination of website and mobile devices may be an effective way to increase participant engagement with the intervention and promote greater behaviour change. Despite the potential of IT-based interventions to change physical activity and dietary behaviours, there have been few IT-based interventions that have been specifically developed for and targeted towards males [9, 20].'

METHODS

3a) CONSORT: Description of trial design (such as parallel, factorial) including allocation ratio

The purpose of this study is to examine the effectiveness of a 9-month web- and mobile phone-based intervention (IT-based) intervention to improve the physical activity, dietary behaviours and health literacy in middle-aged males compared to a print-based intervention [21].   '

3b) CONSORT: Important changes to methods after trial commencement (such as eligibility criteria), with reasons

Not applicable

3b-i) Bug fixes, Downtimes, Content Changes

Not applicable

4a) CONSORT: Eligibility criteria for participants

Males aged 35 to 54 years old who; (1) owned a mobile telephone, (2) had access to the internet, (3) did not have a mobility impairment, (4) resided in the cities of Gladstone or Rockhampton (Queensland, Australia), and (5) were classified as low risk to increase physical activity were eligible to participate in the study. '

4a-i) Computer / Internet literacy

It was not an implicit eligibility criterion (the website was purposefully designed to be very easy and simple to use), as it is likely that most participants would have had some form of Internet literacy, as Internet access was an eligiblity criteria, and all measures were web-based.

4a-ii) Open vs. closed, web-based vs. face-to-face assessments:

To recruit participants, advertisements via local newspapers, trading magazines, face-to-face information sessions with local businesses, and distribution of leaflets and posters to local businesses, medical clinics, and offices of allied health professionals were used. '

'Given participants completed the assessment of outcome measures via online survey... '

4a-iii) Information giving during recruitment

All participants received written and verbal explanation of the project requirements prior to providing consent.'

'To recruit participants, advertisements via local newspapers, trading magazines, face-to-face information sessions with local businesses, and distribution of leaflets and posters to local businesses, medical clinics, and offices of allied health professionals were used. '

4b) CONSORT: Settings and locations where the data were collected

Given participants completed the assessment of outcome measures via online survey,...'

4b-i) Report if outcomes were (self-)assessed through online questionnaires

Given participants completed the assessment of outcome measures via online survey...'

4b-ii) Report how institutional affiliations are displayed

This is not reported in the paper: The recruitment materials clearly indicated they would be participating in research conducted by researchers at the Central Queensland University and the University of Western Sydney. The survey and intervention websites had logo's of the Universities.

5) CONSORT: Describe the interventions for each group with sufficient details to allow replication, including how and when they were actually administered

5-i) Mention names, credential, affiliations of the developers, sponsors, and owners

The authors are the owners and developers of the website, the funder is acknowledged. This is not explicitly mentioned in the manuscript.

5-ii) Describe the history/development process

The ManUp Study was informed by our reviews of published physical activity and dietary interventions for males, our formative research concerning barriers to physical activity and healthy eating behaviours, and our research regarding males' preferences for IT-based interventions [9-11, 20, 21].'

5-iii) Revisions and updating

Intervention content was frozen.

5-iv) Quality assurance methods

Not applicable

5-v) Ensure replicability by publishing the source code, and/or providing screenshots/screen-capture video, and/or providing flowcharts of the algorithms used

No, screenshots can be provided upon request.

5-vi) Digital preservation

No

5-vii) Access

Participants were allocated to intervention arms by project staff using an e-mail with website URL, username and password'

5-viii) Mode of delivery, features/functionalities/components of the intervention and comparator, and the theoretical framework

ManUp intervention

The ManUp Study was informed by our reviews of published physical activity and dietary interventions for males, our formative research concerning barriers to physical activity and healthy eating behaviours, and our research regarding males' preferences for IT-based interventions [9-11, 20, 21]. Both intervention arms provided participants with the same intervention materials and capacity to self-monitor physical activity and dietary behaviours. The IT-based intervention, however, provided participants with the additional ability to receive automated feedback on their progress towards completing their physical activity and dietary behaviours, as well as the ability to interact with other participants on the website [21].

Both interventions arms were provided with educational materials that were specifically designed to present information on the benefits of physical activity and healthy eating, on the volume and type of activity needed to achieve health benefits, and how to achieve the recommended physical activity and dietary behaviours for health benefits. Informed by Social Cognitive Theory and Self Regulation Theory, ManUp "challenges" were developed to change target behaviours by having participants engage in goal setting and self-monitoring behaviours [21-23].

ManUp challenges

The ManUp challenges consisted of six physical activity and a multi-component healthy eating challenge. Each challenge had three different "strengths" (light, mid, full), which varied the duration and the amount of activity or healthy eating that males were asked to achieve in order to complete the challenge. The different activities selected for inclusion were based on those activities frequently performed by Australian males [24]. The ManUp healthy eating challenges were based on achieving a maximum of ten daily healthy eating goals. These goals were informed by the dietary guidelines for Australian adults that promote dietary diversity and encourage the reduction of the intake of saturated fat, salt, alcohol and foods that contain added sugars [25]. Further details on the types of different physical activities and dietary behaviours targeted, the requirements for each challenge, and supporting educational materials are provided in Table 1 and elsewhere [21].

Intervention arms

IT-based intervention arm

Upon completing the baseline assessment participants in the IT-based intervention arm received access to the password-protected ManUp website, which had six main sections [21]. The six sections were: 1) My Profile - where participants could review their current challenges, record their progress towards any current challenges, post personal updates to their profile, schedule future activities, and view information on the groups they were a member of and the list of their 'mates' (online friends on the website); 2) My Progress - where participants could review their progress towards their current challenges; 3) My Mates - where participants could search for online friends and view their mates progress; 4) My Groups - where participants could create a group and view the progress of groups they were part of; 5) My Weight - which provided participants with information on the benefits of achieving a healthy weight, and allowed them to record their height, weight and waist circumference; 6) Information Centre - which provided educational materials related to physical activity and healthy eating, and the challenges [21].

As a form of online social support, participants could comment on their mates' My Profile page. In addition participants could also 'challenge' their mates to complete a physical activity or healthy eating challenge either in a one-on-one basis, or as part of a larger group. A mobile phone web application was developed as an additional tool to facilitate quick and convenient recording of progress towards the ManUp challenges. The mobile phone web application only allowed users to self-monitor behaviour, body weight, and to review progress towards challenge completion. Any participant in the IT-based intervention arm who owned a mobile phone capable of accessing the internet had access to the mobile phone web application.

Print-based intervention arm

Participants in the print-based group were provided with a hard-copy booklet that provided the same educational materials and ManUp challenges as those provided to participants in the IT-based intervention. Participants in the print-based group were not provided with information regarding their peers and, using the provided log sheets, could self-monitor progress and/or successful completion of the ManUp physical activity or healthy eating challenges.'

5-ix) Describe use parameters

The more participants used the intervention the better. Participants were encouraged to use the intervention as much as possible. This is not explicitly mentioned in the paper.
5-x) Clarify the level of human involvement
There was little to no human involvement in either intervention arm.'
5-xi) Report any prompts/reminders used
The IT-based intervention, however, provided participants with the additional ability to receive automated feedback on their progress towards completing their physical activity and dietary behaviours...'
5-xii) Describe any co-interventions (incl. training/support)
A mobile phone web application was developed as an additional tool to facilitate quick and convenient recording of progress towards the ManUp challenges. The mobile phone web application only allowed users to self-monitor behaviour, body weight, and to review progress towards challenge completion. Any participant in the IT-based intervention arm who owned a mobile phone capable of accessing the internet had access to the mobile phone web application. '
6a) CONSORT: Completely defined pre-specified primary and secondary outcome measures, including how and when they were assessed
Outcome measures
Participants completed online surveys at baseline (0 months), 3 months, and 9 months to assess socio-demographic, behavioural, and health literacy outcomes.

Physical activity
Physical activity was assessed using the Active Australia Questionnaire, a valid and reliable instrument that is also sensitive to change in physical activity [26-29]. This questionnaire asks participants to report the duration of recreational and transport walking, moderate and vigorous intensity physical activity in the previous week, and the number of times (sessions) they engaged in these activities. Standard scoring protocols were applied to provide two outcomes; total minutes of physical activity, and the total number of sessions of physical activity [26].

Dietary behaviours
Dietary behaviours were assessed using 19 items adapted from existing instruments used to monitor dietary habits of the Australian population [30, 31]. Two separate items assessed the daily number of serves of fruit and vegetables consumed in the last week, response options ranged from zero serves (don’t eat this food) to ten or more serves. The frequency that red meat, fish, meat products (sausages, salami, meat pies, etc.), cooked cereals, soft drink, chips, takeaway foods, and sweet or savoury foods were consumed in the last week was assessed using response options from rarely/never (don’t eat this food) to more than ten times. The type of milk (whole milk, reduced fat, soy milk, condensed milk, don’t drink milk) and bread white, wholemeal, multigrain, rye, sour dough, other, don’t eat bread) usually consumed was also assessed. Three dietary outcomes were created, type of milk consumed (reduced fat vs. whole milk), type of bread consumed ((higher fibre) wholemeal, multigrain, white with high fibre vs. white, rye, sour dough, other) and an overall index of other dietary behaviours – the dietary score. The dietary score was created by summing the number of serves and number of times the following foods were consumed: fruit, vegetables, red meat, fish, meat products, soft drink, chips, takeaway (take-out) foods, sweet and savoury foods. Several items were reversed score so that higher dietary scores (a better diet) reflected more frequent consumption of healthy food and less frequent consumption of less healthy foods. The dietary score reflected the fact that the ManUp healthy eating challenges focussed on maximising consumption of healthful food and minimising consumption less healthy foods.

Health literacy
Health literacy in relation to physical activity was assessed using the five awareness items from the Active Australia Questionnaire [26]. Using a five-point Likert type scale from strongly agree to strongly disagree, the items assess awareness of the benefits associated with physical activity participation, the intensity and duration required to receive health benefits. Dietary behaviour literacy was assessed using the Nutritional Literacy Survey, a 28-item instrument that assesses participants’ understanding of the type of foods that promote heart health, and the fat and cholesterol content of different foods and portion sizes [32].

IT Platform usage
Usages of the IT-based platform was measured using inbuilt tracking software measuring the number of times a participant logged into the web- and mobile-based platform, made a self-monitoring entry, the type and number of challenges they initiated and completed. '
6a-i) Online questionnaires: describe if they were validated for online use and apply CHERRIES items to describe how the questionnaires were designed/deployed
Though the survey's were not validated for online use, they have been used in online studies many times without problems being reported.
6a-ii) Describe whether and how “use” (including intensity of use/dosage) was defined/measured/monitored
IT Platform usage
Usages of the IT-based platform was measured using inbuilt tracking software measuring the number of times a participant logged into the web- and mobile-based platform, made a self-monitoring entry, the type and number of challenges they initiated and completed. '
6a-iii) Describe whether, how, and when qualitative feedback from participants was obtained
Participants had the option to provide feedback to the researchers (and ethics committee) via e-mail and a form on the website; it was rarely used and not worth reporting on in the manuscript.
6b) CONSORT: Any changes to trial outcomes after the trial commenced, with reasons
Not applicable
7a) CONSORT: How sample size was determined
7a-i) Describe whether and how expected attrition was taken into account when calculating the sample size
Sample Size
Using established methods to estimate sample size [33], the study was powered to detect a 60-minute change in moderate-to-vigorous intensity physical activity per week from baseline to 9 months using an alpha level of 0.05 and a power level of 90%. Based on this calculation, it was estimated that 197 participants would be required. This number was increased, however, to account for the 2:1 allocation of participants in favour of the IT-based intervention arm and the expected drop-out rate of participants (45%) [15, 34]. A higher drop-out rate was used in the current study given the acknowledged difficulty in engaging and retaining males in interventions [9, 20]. As a result, the estimated total sample size was 321; 107 to be allocated to the print-based group and 214 to be allocated to the IT-based group [21]. '
7b) CONSORT: When applicable, explanation of any interim analyses and stopping guidelines
Not applicable
8a) CONSORT: Method used to generate the random allocation sequence
Randomization lists were generated (MJD) using freely available software (www.randomization.com).'
8b) CONSORT: Type of randomisation; details of any restriction (such as blocking and block size)
As IT-based interventions are less frequently examined in male populations [9, 20], the number of participants allocated to the intervention arm was maximised in a 2:1 ratio in favour of the IT-based intervention arm.'

9) CONSORT: Mechanism used to implement the random allocation sequence (such as sequentially numbered containers), describing any steps taken to conceal the sequence until interventions were assigned

As IT-based interventions are less frequently examined in male populations [9, 20], the number of participants allocated to the intervention arm was maximised in a 2:1 ratio in favour of the IT-based intervention arm. Randomization lists were generated (MJD) using freely available software (www. randomization.com).'

10) CONSORT: Who generated the random allocation sequence, who enrolled participants, and who assigned participants to interventions

Randomization lists were generated (MJD) using freely available software (www.randomization.com) and participants allocated to intervention arms by project staff using an e-mail with website URL, username and password. '

11a) CONSORT: Blinding - If done, who was blinded after assignment to interventions (for example, participants, care providers, those assessing outcomes) and how

11a-i) Specify who was blinded, and who wasn't

Participants were blinded to group allocation until after baseline assessments were completed. Given participants completed the assessment of outcome measures via online survey, non-blinding of researchers to participant group allocation was unlikely to bias outcomes. '

11a-ii) Discuss e.g., whether participants knew which intervention was the "intervention of interest" and which one was the "comparator"

Participants were blinded to group allocation until after baseline assessments were completed. ' Given the differences in intervention delivery mode (print vs IT), it was obvious to which group they had been allocated, however it was not specifically mentioned to participants which group the comparator was.

11b) CONSORT: If relevant, description of the similarity of interventions

Print-based intervention arm

Participants in the print-based group were provided with a hard-copy booklet that provided the same educational materials and ManUp challenges as those provided to participants in the IT-based intervention. Participants in the print-based group were not provided with information regarding their peers and, using the provided log sheets, could self-monitor progress and/or successful completion of the ManUp physical activity or healthy eating challenges.'

12a) CONSORT: Statistical methods used to compare groups for primary and secondary outcomes

Analysis

Comparisons between groups at baseline were conducted using generalized linear models and chi-square tests. Comparisons between those participants completing all three assessment points (completers) and those completing less than three assessment points (non-completers) were made on age, education, physical activity, dietary behaviours and health literacy, using t-tests (where parametric assumptions were met) or Mann-Whitney U for continuous variables, and chi-square tests for categorical variables. Generalized linear mixed models use all available data at each time point allowing participants with missing data at follow-up time points to be retained in the analysis. Therefore, generalized linear mixed models with an unstructured covariance matrix were used to examine change over time and differences between intervention arms in physical activity, dietary behaviours, and health literacy outcomes. All analyses were adjusted for baseline age, occupation, and education as these variables are likely to impact upon the physical activity and dietary behaviours of males [35]. Outcomes of the generalized linear mixed model analyses are reported as exponentiated coefficients (b(exp)). To explore the impact of missing data, a sensitivity analysis using baseline observation carried forward (BOCF) for participants with missing data at follow-up time points was performed for physical activity, dietary behaviours and health literacy outcomes; this analysis also adjusted for baseline age, occupation, and education. Comparison of change in physical activity, dietary behaviours and health literacy with and without BOCF revealed only small differences in the magnitude of these outcomes with the exception of consumption of higher fibre bread and low fat milk consumption. For both of these outcomes, the significant time effects present at 3 months in the analysis without BOCF were in the same direction although not statistically significant in the analysis with BOCF. Given these minor differences, only the results from the analyses without BOCF are reported.

Analyses examining the relationship between usage of the IT-platform and change in behaviour within the IT-based intervention arm were conducted using generalized linear models adjusted for age, occupation, education and the baseline level of the outcome examined. The specific model type, link function used for analyses and the total number of observations included are listed in the footnotes of Tables 3, 4 and 5. All analyses followed intention-to-treat principles, and used an alpha level of 0.05. '

12a-i) Imputation techniques to deal with attrition / missing values

Generalized linear mixed models use all available data at each time point allowing participants with missing data at follow-up time points to be retained in the analysis. '

'To explore the impact of missing data, a sensitivity analysis using baseline observation carried forward (BOCF) for participants with missing data at follow-up time points was performed for physical activity, dietary behaviours and health literacy outcomes; this analysis also adjusted for baseline age, occupation, and education. Comparison of change in physical activity, dietary behaviours and health literacy with and without BOCF revealed only small differences in the magnitude of these outcomes with the exception of consumption of higher fibre bread and low fat milk consumption. For both of these outcomes, the significant time effects present at 3 months in the analysis without BOCF were in the same direction although not statistically significant in the analysis with BOCF. Given these minor differences, only the results from the analyses without BOCF are reported. '

12b) CONSORT: Methods for additional analyses, such as subgroup analyses and adjusted analyses

Analyses examining the relationship between usage of the IT-platform and change in behaviour within the IT-based intervention arm were conducted using generalized linear models adjusted for age, occupation, education and the baseline level of the outcome examined. The specific model type, link function used for analyses and the total number of observations included are listed in the footnotes of Tables 3, 4 and 5. All analyses followed intention-to-treat principles, and used an alpha level of 0.05. '

RESULTS

13a) CONSORT: For each group, the numbers of participants who were randomly assigned, received intended treatment, and were analysed for the primary outcome

See Figure 1, the flowchart. The tables also indicate the number of participants analysed for each outcome.

13b) CONSORT: For each group, losses and exclusions after randomisation, together with reasons

Yes, see Figure 1, the flowchart.

13b-i) Attrition diagram

Yes, see figure 4: participant usage of the IT platform.

14a) CONSORT: Dates defining the periods of recruitment and follow-up

Participants (n = 317) were recruited from October 2010 to September 2011'

'Participants completed online surveys at baseline (0 months), 3 months, and 9 months...'

14a-i) Indicate if critical "secular events" fell into the study period

The duration of the study was not long enough for this to be relevant.

14b) CONSORT: Why the trial ended or was stopped (early)

Not applicable

15) CONSORT: A table showing baseline demographic and clinical characteristics for each group

Table 2. Socio-demographic and anthropometric characteristics of participants at baseline.'

15-i) Report demographics associated with digital divide issues

Yes, this is reported in Table 2

16a) CONSORT: For each group, number of participants (denominator) included in each analysis and whether the analysis was by original assigned groups

16-i) Report multiple "denominators" and provide definitions

YES, N values were reported througout the results section, in the tables,
and in the flow chart.

16-ii) Primary analysis should be intent-to-treat

To explore the impact of missing data, a sensitivity analysis using baseline observation carried forward (BOCF) for participants with missing data at follow-up time points was performed for physical activity, dietary behaviours and health literacy outcomes; this analysis also adjusted for baseline age, occupation, and education. Comparison of change in physical activity, dietary behaviours and health literacy with and without BOCF revealed only small differences in the magnitude of these outcomes with the exception of consumption of higher fibre bread and low fat milk consumption. For both of these outcomes, the significant time effects present at 3 months in the analysis without BOCF were in the same direction although not statistically significant in the analysis with BOCF. Given these minor differences, only the results from the analyses without BOCF are reported. '

17a) CONSORT: For each primary and secondary outcome, results for each group, and the estimated effect size and its precision (such as 95% confidence interval)

Yes, these were reported in the tables

17a-i) Presentation of process outcomes such as metrics of use and intensity of use

IT-platform usage

The median number of logins to the IT-platform per week at 3 months and 9 months was 2.00 (IQR = 6.00) and 2.00 (IQR = 6.50), respectively; the average number of logins to the IT-platform at these same time periods was 6.99 (SE = 0.86) and 9.22 (SE = 1.47). Median number of self-monitoring entries per week at 3 months and 9 months was 1.00 (IQR = 20.0) and 1.00 (IQR = 21.5.0) respectively; the average number of self-monitoring entries at 3 months and 9 months was 16.69 (SE = 2.38) and 22.51 (SE = 3.79), respectively. Participants who logged in 2 or more times in the first three months of the intervention made significantly more self-monitoring entries (Median = 18.00, IQR = 38.00) compared to participants logging in less than 2 times (Median = 0.00, IQR = 0.00) (U = 1195.50, P = <0.001). Figure 4 shows the number of users logging in at least once and making at least one self-monitoring entry each week over the intervention period. Following the initial reduction in usage between week 1 and week 3, usage continued to decline throughout the intervention period. No measure of usage of the IT-based platform was associated with any of the physical activity or dietary behaviours examined (Table 5). Use of the 'mate' feature and posts to update of their progress was low; 21 participants used the 'mate' feature and no participants using this feature had more than one mate on the platform; 36 participants used the post feature (minimum = 1 post, maximum = 26 posts).
'

17b) CONSORT: For binary outcomes, presentation of both absolute and relative effect sizes is recommended

Not applicable

18) CONSORT: Results of any other analyses performed, including subgroup analyses and adjusted analyses, distinguishing pre-specified from exploratory

Not applicable

18-i) Subgroup analysis of comparing only users

Such analysis was not undertaken.

19) CONSORT: All important harms or unintended effects in each group

Not applicable, no harms were reported to the investigators.

19-i) Include privacy breaches, technical problems

Not applicable, no privacy breaches or technical problems were reported to the investigators.

19-ii) Include qualitative feedback from participants or observations from staff/researchers

Not applicable

DISCUSSION

20) CONSORT: Trial limitations, addressing sources of potential bias, imprecision, multiplicity of analyses

20-i) Typical limitations in ehealth trials

Males are acknowledged as a hard-to-reach population in the health behaviour intervention literature and this is reflected in the low recruitment rate in this study (approximately 27 participants per month of recruitment). IT-based interventions frequently report low participant retention rates and in this study the overall retention rate at 9 months was 49.2% with a lower retention rate in the IT-based group (46.8%) compared to the print-based group (54.2%) (P = 0.24). This is comparable to previous intervention studies and may therefore not be an issue specific to the target population [58-61]. Consistent with study objectives to improve regular engagement in physical activity and healthy eating, the ManUp study did not focus on weight loss during recruitment which previous studies have promoted in their recruitment [38, 62]. This may have contributed to lower than expected recruitment rates given the prevalence of overweight and obesity in males. Due to logistical constraints it was not possible to assess usage of the print-based materials and self-monitoring behaviour of participants in this intervention arm, this is a limitation of the current study.'

21) CONSORT: Generalisability (external validity, applicability) of the trial findings

21-i) Generalizability to other populations

This study specifically targeted middle aged men, and as such outcomes are not generalizable beyond this group.

21-ii) Discuss if there were elements in the RCT that would be different in a routine application setting

Some of these restrictions were imposed to preserve the integrity of the RCT design which poses interesting design issues for future studies seeking to evaluate the effectiveness of social interaction within RCT designs. These issues include how to foster online social interaction between individuals who do not know each other in real life, or allowing study participant's real life friends to use the platform and maintain the integrity of the trial. '

22) CONSORT: Interpretation consistent with results, balancing benefits and harms, and considering other relevant evidence

22-i) Restate study questions and summarize the answers suggested by the data, starting with primary outcomes and process outcomes (use)

This study examined the relative effectiveness of the ManUp intervention materials delivered by an IT-based intervention platforms compared to a print-based intervention to improve middle aged males' physical activity and dietary behaviours, and health literacy of these behaviours. Analyses revealed significant improvements over time in self-reported minutes and sessions of physical activity and self-reported overall dietary behaviours in both groups. These changes did not significantly differ between participants receiving access to the IT - or print-based intervention materials. '

22-ii) Highlight unanswered new questions, suggest future research

Some of these restrictions were imposed to preserve the integrity of the RCT design which poses interesting design issues for future studies seeking to evaluate the effectiveness of social interaction within RCT designs. These issues include how to foster online social interaction between individuals who do not know each other in real life, or allowing study participant's real life friends to use the platform and maintain the integrity of the trial. '

Other information

23) CONSORT: Registration number and name of trial registry

The study was registered with the Australian New Zealand Clinical Trials Registry (ACTRN12611000081910).

24) CONSORT: Where the full trial protocol can be accessed, if available

Duncan, M.J., et al., Effectiveness of a website and mobile phone based physical activity and nutrition intervention for middle-aged males: trial protocol and baseline findings of the ManUp Study. BMC Public Health, 2012. 12: p. 656.

25) CONSORT: Sources of funding and other support (such as supply of drugs), role of funders

Acknowledgements.
Queensland Health provided funding to conduct this project and to develop all intervention materials. This manuscript was partially supported by the CQUniversity Health CRN.'

X26-i) Comment on ethics committee approval

All participants received written and verbal explanation of the project requirements prior to providing consent, provided informed consent prior to participation in the study, and the Central Queensland University (H10/07-131) and the University of Western Sydney Human Research Ethics Committee approved the study (H8605). The study was registered with the Australian New Zealand Clinical Trials Registry (ACTRN12611000081910).'

x26-ii) Outline informed consent procedures

All participants received written and verbal explanation of the project requirements prior to providing consent, provided informed consent prior to participation in the study, and the Central Queensland University (H10/07-131) and the University of Western Sydney Human Research Ethics Committee approved the study (H8605). The study was registered with the Australian New Zealand Clinical Trials Registry (ACTRN12611000081910).'

X26-iii) Safety and security procedures

Males aged 35 to 54 years old who; (1) owned a mobile telephone, (2) had access to the internet, (3) did not have a mobility impairment, (4) resided in the cities of Gladstone or Rockhampton (Queensland, Australia), and (5) were classified as low risk to increase physical activity were eligible to participate in the study. '

X27-i) State the relation of the study team towards the system being evaluated

The authors were able to develop the intervention due to funding from an external funding body:

'Acknowledgements.
Queensland Health provided funding to conduct this project and to develop all intervention materials. This manuscript was partially supported by the CQUniversity Health CRN.'
